# Remote assessment of infant development: Is universal screening of early motor milestones within reach?

**DOI:** 10.1111/dmcn.70241

**Published:** 2026-03-13

**Authors:** Deirdre Murray

**Affiliations:** ^1^ Department of Paediatrics and Child Health, College of Medicine and Health, University College Cork, INFANT Maternal and Child Health Research Centre, Cork University Hospital Cork Ireland

## Abstract

**This commentary is on the original article by Airaksinen et al. on pages** 1440–1449 **of this issue.**

Developmental assessment of young children is challenging. Detailed assessment with a trained therapist is prohibitively expensive for widespread screening, reserved only for high‐risk children or those in whom early concern is raised. Thus, even in high‐income countries, only 1% to 2% of all children will be assessed using detailed and objective measurements. The other 98% may, at best, be screened using parental questionnaires, with inherent difficulties. Parents can generally be relied upon to accurately list the motor skills achieved; however, the quality of movement and normality of tone and posture is more difficult to capture. Hence a wide gap exists between universal parental questionnaires and standardized face‐to‐face developmental or neurological assessment. The Ages and Stages Questionnaires are the most universally recommended, but have low sensitivity for mild delay, particularly in typically developing cohorts.^1^

Airaksinen et al. raise the question as to whether technology can fill this gap and allow reliable universal early motor screening.^2^ The authors present data on the use of a wearable suit for the automated assessment of motor development in young children.^2^ Families of 42 infants were given wearable suits with in‐built sensors (Motor Assessment of Infants with a JUmpsuit [MAIJU]) which tracked their movements and postures for an average of 2 hours. Automated reports of posture and movement were generated (BABA infant motor score) with a high correlation with an in‐clinic or video‐assessed Alberta Infant Motor Scale.

Why is early screening of motor milestones important? These milestones are surrogate markers of overall brain and nervous system development. Motor delay may be the first sign of general developmental delay and often precedes social, behavioural, and cognitive delay. Equally, aberrant motor development may herald evolving signs of cerebral palsy (CP). The success of early CP screening in high‐risk infants through the combination of imaging, general movements assessment, and the Hammersmith Infant Neurological Examination remains limited to infants with clear risk factors at birth.^3^ We know that at least 50% of CP cases are born outside of this risk profile and their aberrant motor development must be picked up by parents who then seek out trained professionals; a gap which results in delayed detection and delayed intervention. Technology might also help to fill this gap.

Videos of an infant's early movements provided by carers via smartphone‐based applications can be accurately analysed by remote assessors and are comparable to in‐person general movements assessment. However, the manpower and expertise required for remote human assessment is still prohibitive for universal screening. This has driven researchers to explore the ability of artificial intelligence (AI) to replace the remote assessor and to risk‐stratify for CP. Results are promising but currently limited to a few small populations.^4^ Collaboration between these groups is vital to allow validation of these promising AI models, and to ensure that they are suitable for use in all populations, not limited to specific cultural norms. Large libraries of video and sensor‐based data from geographically and culturally diverse populations will be required. Data will need to be analysed in a way which is compliant with ethical and data‐sharing regulations to ensure protection of the children involved.^5^ Whilst early general movements assessments are a snapshot at a specific phase in early development, serial recordings of movement and posture over the first year of life may be required to fulfil the promise of early motor screening for all. The MAIJU suit is not designed for CP screening and has not yet been validated in a high‐risk group. It is unlikely to detect subtle asymmetries of tone. However, it offers the potential of early serial tracking of movement and posture and is a good step in the right direction.

## Data Availability

Not required.
